# The Dynamics of Health and Return Migration

**DOI:** 10.1371/journal.pmed.1001046

**Published:** 2011-06-21

**Authors:** Anita A. Davies, Rosilyne M. Borland, Carolyn Blake, Haley E. West

**Affiliations:** Migration Health Division, Department of Migration Management, International Organization for Migration, Geneva, Switzerland

## Abstract

In the final article in a six-part *PLoS Medicine*; series on Migration & Health, Anita Davies and colleagues from the International Organization for Migration (IOM) discuss the specific health risks and policy needs associated with return migration

**Summary Points**

- The increasing importance and complexity of migration globally also implies a global increase in return migration, and thus an increased interest in the health of returning migrants.
- The health of returning migrants is impacted by the cumulative exposure to social determinants and risk factors of health during the migration process, during the return movement, and following return.
- Circular migration often occurs among the diaspora, which can result in the transfer of knowledge and skills that contribute to development, including health system strengthening.
- Migrants with dual nationality often return to countries with better health services than their country of origin when they are sick and can not get care at home.
- To maintain and improve the health of returning migrants, multi-sectoral policies at global and national levels should facilitate access to appropriate and equitable health services, social services, and continuity of care across and within borders.

**This is one article in a six-part** ***PLoS Medicine*** **series on Migration & Health.**

## Introduction

Return migration is part of the migration process and refers to the act of going back to a place of origin, whether within the territorial boundaries of a country, as in the case of returning internally displaced persons (IDPs); or from a host country to an origin country, as in the case of refugees, asylum-seekers, and international migrant workers [1]. Within the overall complexity of migration, return migration happens in a range of contexts. While migration in and of itself is not necessarily a risk to health, the conditions surrounding the process can increase health vulnerabilities [2]. Factors related to the migration process, such as reasons for migrating, type of travel, length of stay, and legal status can act as determinants of a migrant's health [3].

Return migration and health has received little attention in policy and research. This article will focus on the risk factors and social determinants of health during all phases of migration that can impact the health of returnees. It will highlight the diversity of returning migrants and illustrate through examples how return migration can influence the health of individuals and populations. The paper will conclude with policy recommendations for healthy return migration.

## The Health of Returning Migrants

The health status of returning migrants reflects the accumulation of health consequences related to the conditions of the migration process, including the return phase. The health of returnees is closely related to the social determinants of and risk factors for health, as well as to immigration and labour policies influencing the migrant's access to health services.

Migrants return home with a wide range of health needs. Some returnees arrive healthy, as in the case of migrants who have been able to get good jobs in destination countries and have had access to appropriate health and social services. This is often the case with healthy retired professionals who return with money and contribute to development in their countries of origin. However, during their process of aculturisation in the destination country, some migrants may have acquired unhealthy lifestyles that increase their risk for non-communicable diseases such as cardiovascular disease or diabetes. Only wealthy returning migrants will be able to afford the available health services and go abroad for health services unavailable in their country of origin. Research has shown returnees with dual residency are more likely to return to their alternate country of residence to access health care and social benefits when not available in countries of origin [4].

Migrants who receive low wages and live in poor housing, eat unhealthy food, and have difficulty accessing health services may have been exposed to other risk factors that promote poor health. These migrants often return home less healthy than when they left. This group of returnees may need health care that does not exist or which they can not afford. In extreme situations, migrants are forced to return home because of ill-health, chronic diseases, and terminal illnesses, as they often prefer to retire or die in their place of origin [5]–[7].

Three cases—migrant workers, returning IDPs, and qualified health professionals—provide insights into the health and policy needs associated with the return phase of migration.

### Migrant Workers

Much of migration is linked to the search for a better life, including economic opportunities. An estimated 47% of international migrants are labour migrants [8]. Though migrant workers' remittances are now three times as large as official development assistance, the health of returning migrant workers (including those with formal contracts and legal migration status) is often not prioritised [9]. While some countries have made important strides in working with international migrant workers *before* they migrate [10], facilitating access to health services upon *return* remains a critical gap.

Migrant workers return to their countries (or communities) of origin in a variety of ways. Labour migrants may return through legal channels following formal temporary labour migration schemes organised between governments or companies [11]. Other migrant workers return irregularly, following undocumented or informal work in a destination setting. Irregular or stranded migrant workers may accept return support through assisted voluntary return programmes [1], while others may be detained in destination countries and forcibly deported back to their countries of origin by governments. Each of these modes of return is associated with different health risks, which can directly affect the health outcomes of a migrant worker during and following the return.

Irregular migration—movement that takes place outside the regulatory norms of the sending, transit, and receiving countries [1]—poses specific challenges to health systems and individual health. Irregular migrant workers do not benefit from existing legal protections that apply to regular migrants and formal workers [12],[13]. In addition to the health risks experienced while working, such migrants may be exposed to additional health risks during return. If travel is clandestine, irregular migrant workers may face extreme conditions during the journey with specific health consequences (e.g., dehydration, physical injury, and exposure to communicable diseases) [14]. Irregular migrants are also vulnerable to abuse during their attempts to cross land and sea borders, and migrant women in particular often face sexual violence [15]. In some cases, migrant workers may fall into the hands of human traffickers for the purpose of forced labour or sexual exploitation. Trafficked persons often experience a range of abuse, deprivation, and violence, resulting in serious health consequences that persist after the exploitation has ended [16]. For survivors of trafficking who have returned to their community of origin, the psychological distress in particular can have long-term consequences on their health [17].

Upon return, there is evidence that health and referral systems are often lacking [18]. Irregular migrant workers forcibly returned by governments may not receive adequate health assistance during detention prior to movement, or referral to health services upon arrival [19]. A formal seafarer returning may be debriefed upon return, but may not interact with the health system until medically assessed prior to the next contract, despite potential health needs related to the recently completed migration. Though some migrant workers seek services for health problems related to their time away from home, some delay seeking health care, sometimes leading to poorer health outcomes. A migrant who is detained and deported may be received by civil society or reception centres in exceptional cases, but is often processed by migration authorities and does not undergo a health assessment [20]. This occurs despite the fact that many irregular migrant workers may have experienced some form of abuse, or faced critical health conditions during the migration process. Even migrant workers who have already received some health support and are assisted to return home through voluntary return programmes may have limited health assistance available once they have returned. Finally, in some cases, migrant workers may be forcibly returned due to their health status, such as when migrants are deported based on HIV status, as illustrated in Box 1.

**Box 1. Case Study: Forced Return of Migrant Workers Based on HIV Status**

- Migration to Arab states for domestic work is common among female migrants from countries in Asia [21]. As with other groups of migrant workers, these women play a critical role in the economies of their home countries through remittances. Yet, little protection exists during the migration process to prevent them from exposure to health risks such as sexual violence, exploitation, and dangerous travelling and living conditions: “Unsafe migration, duress in the workplace, sexual exploitation (both in the home and host country), lack of legal coverage, and limited or no access to health or social services tends to make women migrants, especially in the domestic sector, particularly vulnerable to HIV” [21].
- Globally, there are currently 29 countries around the world that deport migrants who are HIV positive due to their status [22]. Destination countries often require yearly medical screenings for migrant workers which may include a mandatory HIV test. Many times the migrant may not speak the local language and may not realize what they are being tested for. If they do test HIV positive, the migrant is frequently not told the test results, but only informed that they have failed the exam and are to be detained or immediately deported [23]. Once a domestic worker has tested positive for HIV, it is nearly impossible to re-migrate for work due to discriminatory HIV-related entry, stay, and residency restrictions in the legislation [24]. Deportation thus means a loss of income for the individual and family in both the short and long term.
- For individuals found to be HIV positive, the conditions before and during their forced return combined with the quality of care and treatment services in their communities of origin can greatly affect their health. Detained without counselling or treatment and then possibly returned to an origin country with limited capacity to treat, a domestic worker who is HIV positive has limited, if any, options for managing this chronic condition.

### Internally Displaced Persons

The living conditions of IDPs in community or camp settings can have an influence on their health status upon return. Exposure to risk factors may lead to chronic diseases, mental health conditions, and infectious diseases that can persist upon return to their places of origin [25],[26]. Most countries affected by internal displacement also experience a breakdown of health services. During a mass return of IDPs, weakened health services have difficulty coping with the increased demand, given the lack of national financial and human resources for the health sector [27].

Returnees from areas that do not have the same endemic infectious diseases in their places of origin, such as malaria, may lack immunity and are susceptible to these diseases upon return [28],[29]. Migrants may also return with diseases that are not endemic to their places of origin. This will pose challenges to health workers who are not familiar with these new diseases, and can not diagnose and treat appropriately. Medication to treat these new diseases may not be readily available.

Returning migrants, particularly women and children who have been exposed to sexual violence during displacement, are often stigmatised and may not receive appropriate treatment upon return, because of high out-of-pocket payments, or because the appropriate services and qualified health workers are simply not available [30],[31]. Returning to a supportive social network, language, and social norms and a health system that the returnee understands often contributes to improve the returnees' physical, mental, and social well-being. Initiatives to facilitate the return of qualified health professionals to strengthen health systems have been implemented through collaborations between countries of origin and destination, and organisations such as the International Organization for Migration (IOM) [32] (see Box 2).

**Box 2. Returning Migrant Health Workers to Strengthen Health Systems**

- The permanent or temporary return of migrant health professionals is essential for rebuilding and strengthening weak health systems to meet the health needs of returning and host populations in politically stable situations.
- The International Organization for Migration (IOM) has developed a framework called the Migration for Development in Africa (MIDA) health initiative to facilitate the return of health professionals in the diaspora for health system strengthening [32]. A MIDA health initiative has been implemented in Ghana where health professionals have returned from Europe to strengthen health service delivery and capacity development. The principles of MIDA have also been used to strengthen health systems in crisis situations and post conflict situations.

## Return Migration Health Policies and Strategies

Global or regional policies and strategies to provide adequate health and social services to returning migrants do not exist. Both destination and origin countries have a role to play in developing and implementing repatriation policies and strategies that include access to health and social services upon return. At the national level, Mexico has developed a strategy that facilitates the repatriation of seriously ill patients from the United States and ensures treatment in Mexico [34]. Furthermore, few countries have health insurance schemes that migrants can benefit from upon return to their countries of origin. There are lessons to be learnt from the Philippines, where overseas Filipino workers pay into a health insurance that allows them to access health care upon return to the Philippines [35].

Some countries have bilateral agreements between destination and origin countries to facilitate assisted voluntary return, providing migrants' access to health services as part of reintegration. Migrants that participate in such programmes may also be assisted with income-generating activities to promote self sustainability upon return.

Countries of origin are becoming more aware of the number of returning migrants, and in cases of mass return, as in the case of crisis in North Africa in 2011, many governments and international organisations such as the Red Cross/Red Crescent provide a health assessment upon return and referral to appropriate services (see Box 3).

**Box 3. Case Study: Return Migration and Health in Post-Conflict Sri Lanka [33]**

- Since the end of the conflict in May 2009, the Government of Sri Lanka, IOM, and other stakeholders have collaborated to assist about 280,000 internally displaced persons living in IDP camps. Interventions were carried out within the camps and also in the return migration process. Logistics were put into place to assist the transportation of more than 100,000 persons to their districts of origin in the northern and eastern war-affected region of Sri Lanka. In terms of health care, the priority was health system strengthening and addressing social risks and vulnerabilities to ill health.
- Post-conflict health system recovery was carried out by building the capacity of local government and health personnel in return areas. IOM facilitated health worker mobility to areas of return, provision of ambulances for emergency referrals, building and rehabilitation of referral hospitals and primary health care centres, supporting mobile medical clinics to reach remote return areas, as well as training of health personnel in disease surveillance and clinical management.
- On the prevention and community health level and in collaboration with the government and other stakeholders, IOM performed rapid assessments to ensure areas of return were safe (i.e., de-mining). IDPs were provided with shelter assistance, water and sanitation systems, and assistance to launch income-generating activities.
- This example illustrates the importance of a multi-disciplinary approach, which maintains the link between humanitarian and development work, in order to address the health risks and vulnerabilities of returning IDPs.

## Policy Recommendations

Recommendations to ensure that migrants are healthy upon return and remain healthy in their communities of origin include the following:

- Development of national, regional, and international policies and strategies to anticipate and address the needs of returning migrants. Policy and legislation at the international, regional, and national levels should facilitate continuity of care across and within borders, for instance, pensions accessible from abroad, transferable pensions, and treatment schemes, as well as harmonised treatment protocols.
- Health assessments to identify urgent needs and facilitate referral should be made available to returning migrants, including those participating in mass return, assisted voluntary return, at border areas and reception centres for deportees, and irregular migrants.
- Health data and records should be transferable under the guidance of international data protection and confidentiality standards and laws to enable referral and continuity of care across borders.
- Efforts should be made to improve research and data collection on the number of emigrating and immigrating migrants to provide information for health and development policy-makers to provide adequate health and social services for all returnees.

## Conclusions

Despite the significant impact of the return phase on the health of migrants, there is a gap in policies and programmes to address the needs of returning migrants. Given the increase in return migration worldwide, there is a need for coherent multi-sectored migrant health global and national strategies. The health of migrants has recently gained interest on the global agenda as is seen in the adoption of the resolution on the health of migrants (WHA 61.17) during the 61st World Health Assembly in 2008 [36], as well as the recent Global Consultation on the Health of Migrants [37]. These stepping stones in migrant health call for the improvement of access to health and social services for migrants at all phases of migration, without forgetting the return phase. It is in the best interest of all countries to promote the health of returning migrants, irrespective of whether they are a country of origin, transit, or destination.

## Supporting Information

Text S1 **Alternative language Summary Points.** Translated into French by Carolyn Blake and Spanish by Rosilyne M. Borland.
(DOC)

## Abbreviations

IDP: internally displaced person
IOM: International Organization for Migration
