# Supplementary material for: The Dynamics of Health and Return Migration
Source: PLoS Med. 2011 Jun 21;8(6):e1001046. doi: 10.1371/journal.pmed.1001046 (PMC3124523; doi:10.1371/journal.pmed.1001046)
Supplement: Text S1 — Alternative language Summary Points. Translated into French by Carolyn Blake and Spanish by Rosilyne M. Borland. (DOC) [file pmed.1001046.s001.doc]

**Summary points**

- The increasing importance and complexity of migration globally also implies a global increase in return migration, thus an increased interest in the health of returning migrants.
- The health of returning migrants is impacted by the cumulative exposure to social determinants and risk factors of heath during the migration process, during the return movement and following return.
- Circular migration often occurs among the diaspora for the transfer of knowledge and skills to contribute to development including health system strengthening.
- Migrants with dual nationality often return to countries with better health services than their country of origin when they are sick and can not get care at home.
- Multi-sectoral policies at global and national level should facilitate access to appropriate, equitable health and social services and continuity of care across and within borders to maintain and improve the health of returning migrants.

**Résumé**

- L’importance et la complexité croissante de la migration dans le monde, implique également un accroissement dans la migration de retour, et ainsi un intérêt grandissant pour la santé des migrants retournant dans leur lieu d’origine.
- Durant tout le processus migratoire de retour, incluant le mouvement et l’arrivée, la santé des migrants est influencée par une exposition cumulative à des déterminants sociaux et des facteurs de risque de santé.
- La migration circulaire a fréquemment lieu parmi la diaspora, et participe au transfert de connaissances et de compétences, contribuant ainsi au développement socio-économique et au renforcement des systèmes de santé.

- Les migrants avec double-nationalités nécessitant des soins médicaux, utilisent souvent la possibilité de retourner dans le pays offrant les meilleurs services de santé.

- Des politiques multisectorielles au niveau national et global devraient être développées, afin de garantir un accès équitable à des services sociaux et de santé de qualité. De plus, la continuité des soins à travers et à l’intérieur des frontières nationales devrait être une priorité afin de maintenir et améliorer la santé des migrants retournant dans leur lieu d’origine.

**Resumen**

- La creciente importancia y complejidad de la migración a nivel mundial implica también un aumento global de la migración de retorno, por lo tanto un mayor interés en la salud de los migrantes que regresan
- La salud de los migrantes que regresan se ve afectada por la reiterada exposición a los determinantes sociales y los factores de riesgo para la salud durante el proceso de migración, el movimiento de retorno y tras su llegada
- La migración circular se produce entre la diáspora para la transferencia de conocimientos y habilidades para contribuir al desarrollo, incluyendo el fortalecimiento del sistema de salud
- Los migrantes con doble nacionalidad regresan con frecuencia a los países que tienen mejores servicios de salud que los de su país de origen cuando están enfermos y no pueden recibir atención en su país de residencia.
- Políticas multisectoriales a nivel mundial y nacional deberían facilitar el acceso a la servicios de salud y servicios sociales, adecuados , equitativos así como la continuidad de la atención a través y dentro de las fronteras para mantener y mejorar la salud de los migrantes que regresan
